# Xanthine Oxidoreductase Reference Values in Platelet-Poor Plasma and Platelets in Healthy Volunteers

**DOI:** 10.1155/2015/341926

**Published:** 2015-01-20

**Authors:** Elżbieta Cecerska-Heryć, Anna Jesionowska, Szupiluk Klaudyna, Siewierska Katarzyna, Mączka Dominika, Pawlak Dominika, Urbańska Marta, Barbara Dołęgowska

**Affiliations:** Department of Laboratory Diagnostics and Molecular Medicine, Pomeranian Medical University of Szczecin, Powstancow Wielkopolskich 72, 70-111 Szczecin, Poland

## Abstract

*Introduction.* Xanthine oxidoreductase (XOR) is an enzyme belonging to the class of hydroxylases. XOR is stated, inter alia, in the kidneys, liver, and small intestine as well as in leukocytes and platelets and endothelial cells of capillaries. Its main role is to participate in the conversion of hypoxanthine to xanthine and the uric acid. It occurs in two isoforms: dehydrogenase (XD) and oxidase (XO), which is considered one of the sources of reactive oxygen species.* Aim of the Study.* Determination of reference values of xanthine oxidoreductase activity in PPP and platelets.* Materials and Methods. *Study group consisted of 70 healthy volunteers. The isoform activities of xanthine oxidoreductase were determined by kinetic spectrophotometry.* Results.* A statistically significant difference between the activity of the XOR in PPP and platelets (*P* < 0.001). The highest activity of XO was found in both PPP and blood platelets. Significant differences between the activity of the various isoforms in PPP (*P* = 0.0032) and platelets (*P* < 0.001) were also found.* Conclusions.* The healthy volunteers showed the highest activity XO (prooxidant) and the lowest XD (antioxidant), which indicates a slight oxidative stress and confirmed physiological effects of XOR.

## 1. Introduction

Xanthine oxidoreductase (XOR) is a molybdate hydroxylase, which is to catalyze the oxidation of hypoxanthine to xanthine and xanthine to uric acid. It comes in two mutually converting alternative isoforms: xanthine dehydrogenase (XD), expressed in vivo as well as in healthy tissue of the parent, and xanthine oxidase (XO), generated by the posttranslational modification of the XD or by oxidation of cysteine residues and also limited proteolysis, which plays a dominant role in cells and tissues during trauma [1–3]. Both isoforms act in opposition to each other. XD feedstock for the oxidation of the oxidized form NAD^+^ also has a preference for the NAD^+^ as a cosubstrate, also has the ability to react with O_2_ [4], and also catalyzes a reaction in which uric acid (UA) is synthesized. It has demonstrated a protective effect in the body, to trap the reactive oxygen species (ROS), and acts as an antioxidant [5].

On the other hand, XO is unable to bind NAD^+^, and O_2_ is used in the reaction associated with increased synthesis of ROS and RNS (reactive forms of nitrogen), which is an important relay inflammatory response through the activation of the complement system or modulation of endothelial P-selectin expression on the cell surface [6]. It is xanthine oxidase that is considered to be the main source of reactive oxygen species in a variety of clinical settings as well as under conditions of hypoxia and reperfusion and organ failure [1].

Xanthine oxidoreductase itself can act in two ways: in the presence of NAD^+^ as dehydrogenase and molecular oxygen as an oxidase. The ability to rapidly convert XOR of antioxidant to the oxidant, the various kinds of tissue damage, is an essential element for rapid innate immune response, a preferred example in bacterial or fungal infection [7].

Xanthine dehydrogenase, reactive oxygen intermediate form called (XDO), and its presence were assumed since the 70s of the last century. It reacts with both NAD^+^ and O_2_, with the proviso that it retains greater affinity for NAD^+^. The activity of both XD and XDO is regulated by the [NAD^+^]/[NADH + H^+^] [8]. The isolation of the intermediate isoform is irrelevant molecular, but the indication of its activity, allows to follow the process of transforming the dehydrogenase to oxidase isoforms [9].

The literature reports cannot be found, on the activity of the various isoforms of xanthine oxidoreductase platelet-poor plasma (PPP) and platelet-rich plasma (PRP) in healthy controls. It has been shown, however, that the activity of the XOR in the serum of healthy individuals is very low, which corresponds to the production of less than 4 O_2_/mL plasma nmol/min (calculated as the reduction of ferricytochrome C ROS). However, the growth is a characteristic of various pathological conditions such as viral hepatitis, autoimmune rheumatic diseases, chronic kidney disease, type 2 diabetes, and schizophrenia [10, 11]. Important physiological XOR and forecasting in the case of many diseases such as cancer require knowledge of xanthine oxidoreductase reference standards in PPP and in platelets, which is also the aim of this work.

## 2. Materials and Methods

The material was collected in the morning from 70 healthy volunteers fasted, among whom were 48 women and 29 men. After downloading the material, volunteers were asked to fill out a survey on the general information about the patient and his health condition. In addition to marking the XOR and its isoforms, blood counts and biochemical tests were also performed, in all volunteers. Patients were divided into two groups above and below 30 years of age in order to perform further analyses (detailed information about the volunteers: Table 1).

The determinations were made in PPP and in platelet lysates (Table 2). The plasma was thawed at room temperature and then centrifuged (10 min, 4°C, and 3824 g). The plates were thawed at room temperature and then centrifuged (10 min, 4°C, and 3824 g) to give a clear lysate [12, 13].

### 2.1. Xanthine Oxidoreductase Activity Determination of Plasma and Platelets

Reagents Trizma base; NAD^+^; xanthine; CuSO_4_∗5  H_2_O were purchased from Sigma Aldrich (Poznan, Poland). Determinations were performed using a UV/VIS Lambda 40P (Perkin Elmer). Extinction changes were recorded for 5 minutes at 30°C. The enzymatic activity was measured as formation of uric acid and NADH (increases in A_340_ and A_302_) and expressed in mU/mL (milliunits per milliliter). The enzymatic activity was calculated taking into account the initial rates of reaction. Uric acid formation was measured at 302 nm (isoforms XDO and XO) because its absorbance is still high there, whereas changes in NAD^+^ concentration do not contribute. During the calculation of isoforms activity of xanthine oxidoreductase included molar extinction coefficients: NADH + H^+^ *ε*340 = 6.22 × 103 L mol^−1^ × cm^−1^; NADH + H^+^ *ε*302 = 2.30 × 10^3^ × mol^−1^ cm^−1^ [8, 9].

### 2.2. Xanthine Oxidoreductase Activity Determination of PPP

Isoform dehydrogenase (XDH) was determined by measuring the increase in the extinction of the test sample (500 mM NAD^+^, 50 mM buffer Tris/HCl: pH 8.0, and 100 mM xanthine; PPP) at a wavelength of 340 nm against the blank (500 mM NAD^+^, buffer 50 mM Tris/HCl: pH 8.0, and 100 mM xanthine).

Intermediate isoform (XDO) was determined by measuring the increase in the extinction test sample (500 mM NAD^+^, 50 mM Tris buffer/HCl: pH 8.0, and 100 mM xanthine; PPP) at a wavelength of 302 nm, against the blank (500 mM NAD^+^, buffer 50 mM Tris/HCl: pH 8.0, and 100 mM xanthine).

Isoform oxidase (XO) was determined by recording an increase in the extinction test sample (500 mM NAD^+^, 50 mM Tris buffer/HCl: pH 8.0, and 100 mM xanthine; PPP) at a wavelength of 302 nm against the blank (500 mM NAD, buffer 50 mM Tris/HCl: pH 8.0, and 100 mM xanthine) [8, 9].

### 2.3. Determination of Xanthine Oxidoreductase Activity Platelets

**Figure 1:**
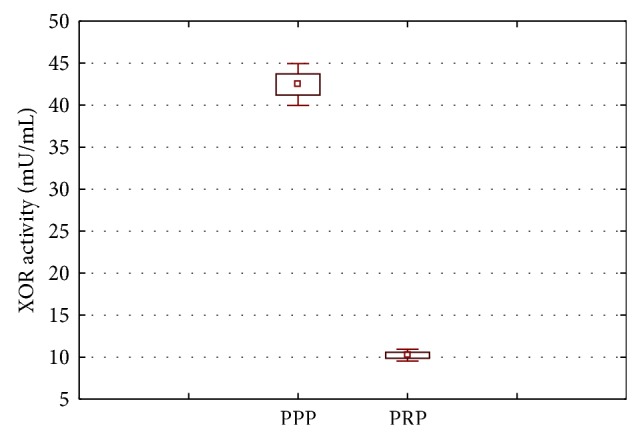
Comparison of activity in PPP XOR and platelets (*P* < 0.001). Whiskers show the value of the lower and upper quartile.

**Figure 2:**
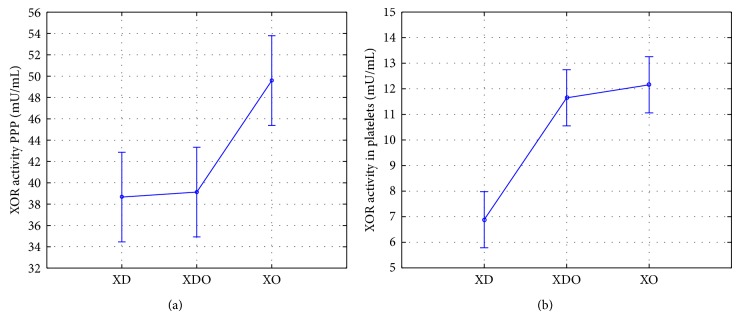
ANOVA analysis of the relationship in the activity of the various isoforms in xanthine oxidoreductase PPP (a) (*P* = 0, 0032) and platelets (b) (*P* < 0, 001) (mean ± 95% CI). XDH: dehydrogenase isoform; XDO: isoform dehydrogenase-oxidase (indirect); XO: oxidase isoform.

Isoform dehydrogenase (XDH) was determined by measuring the increase in the extinction of the test sample (250 mM NAD^+^, 50 mM buffer Tris/HCl: pH 8.0, 50 mM xanthine, and 5 mM CuSO_4_; platelet lysate) at a wavelength of 340 nm against the blank (250 mM NAD^+^, 50 mM buffer Tris/HCl: pH 8.0, 50 mM xanthine, and 5 mM CuSO_4_) (Figures 1 and 2).

Intermediate isoform (XDO) was determined by measuring the increase in the extinction of the test sample (250 mM NAD^+^, 50 mM buffer Tris/HCl: pH 8.0, 50 mM xanthine, and 5 mM CuSO_4_; platelet lysate) at a wavelength of 302 nm, compared to the blank (250 mM NAD^+^, 50 mM buffer Tris/HCl: pH 8.0, 50 mM xanthine, and 5 mM CuSO_4_).

Isoform oxidase (XO) was determined by recording an increase in the extinction test sample (500 mM NAD^+^, 50 mM Tris buffer/HCl: pH 8.0, and 100 mM xanthine; PPP) at a wavelength of 302 nm against the blank (250 mM NAD^+^, buffer 50 mM Tris/HCl: pH 8.0, 50 mM xanthine, and 5 mM CuSO_4_) [8, 9].

### 2.4. Statistical Analysis

The results were statistically analyzed. The assessment of normality of distributions was performed by Shapiro-Wilk test. To assess the differences between the studied parameters, *t*-tests for related and unrelated variables and one-way ANOVA were used. Analysis of variance was performed using *F*-test (for the two series of analyses of variance) and Levene's test (for homogeneity of variance multiple series). The assumptions required for the application of analysis of variance (normality of distribution and homoscedasticity) are not violated in a way that could disrupt the reliability of statistics *F*. Chi-square test was used to analyze qualitative data.

Statistical study of the results was performed using the statistical program STATISTICA GB 10 (StatSoft). The level of statistical significance was taken as *P* < 0.05.

## 3. Results

There is a statistically significant difference (*P* < 0.001) between the activity of xanthine oxidoreductase PPP in plasma and its activity in platelets (PRP). Higher activity in all isoforms oxidoreductase PPP compared to its activity in platelets was also demonstrated. Highest activity of XO and XDH in both serum and the PPP and PRP isoforms was observed best. The differences in the activity of the individual isoforms are statistically significant and are, respectively, PPP *P* = 0.0032 and the platelet *P* < 0.001.

### 3.1. Xanthine Oxidoreductase Activity of Patient Age and Gender

There was no effect of gender on patient activity of xanthine oxidoreductase. There was no effect of age on the enzyme activity, while in the case of oxidoreductase activity in PPP close correlation was statistically significant (*P* = 0.055), wherein the substantially higher activity of the oxidoreductase occurred among people over 30 years of age (Figure 4).

### 3.2. Reference Value Calculation Xanthine Oxidoreductase

XOR reference values and its isoforms were determined in the range of 2.5–97.5 percentile obtained by the spectrophotometric determinations of enzyme activity (Table 3).

## 4. Discussion

Xanthine oxidoreductase activity in human and animal tissues has been quite extensively studied by many researchers. On the basis of these results, it is determined that the highest activity of the XOR is present in the liver and intestine, and ambiguous results were obtained with serum, skeletal muscle, brain, and heart. Such studies were also carried out for various kinds of diseases [14]. For example, Aliciguzel et al. showed that there was no significant difference in the activity of XDH and XO in the liver of rats with early and late diabetic group versus control. Compared to the control there was no significant difference in the activity of XO in the heart, kidney, and brain. However, the XDH activity in these tissues was significantly higher in diabetic rats than in late diabetic rats with early diabetic group or control [15].

In the literature, it is hard to find, in turn, information on the activity of XOR and its isoforms in platelet-poor plasma and platelets in healthy volunteers. Shamma et al. showed that the activity of the XOR in the serum of individuals not suffering from any disease is very low, which corresponds to the production of less than 4 O_2_/mL plasma nmol/min (calculated as the reduction of ferricytochrome C ROS). However, the growth is a characteristic of various pathological conditions such as viral hepatitis, autoimmune rheumatic diseases, chronic kidney disease, type 2 diabetes, and schizophrenia [11].

Other pieces of information on the activity in healthy individuals XOR in the material used in the above study were not found. Quite often is undertaken on the activity of the enzyme in the plasma of patients suffering from, for various kinds of diseases.

**Figure 3:**
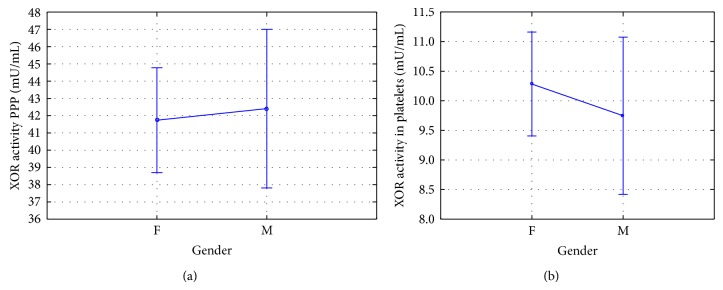
Based on the ANOVA analysis of healthy volunteers between sex and xanthine oxidoreductase activity in PPP (a) and platelets (b) (mean ± 95% CI). The activity of xanthine oxidoreductase in the PPP according to gender (*P* = 0.811); xanthine oxidoreductase activity in platelets according to gender (*P* = 0.507).

**Figure 4:**
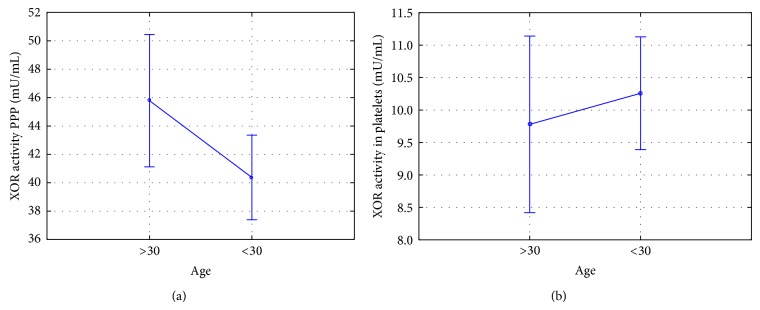
ANOVA analysis of the effects of age on healthy volunteers xanthine oxidoreductase activity in PPP (mean ± 95% CI). The activity of xanthine oxidoreductase in PPP in different age groups (*P* = 0.055); xanthine oxidoreductase activity in platelets in the different age groups (*P* = 0.559).

In this study, a higher activity of all isoforms XOR in PPP compared to platelets is showed. It was also a statistically significant difference in the activity of the enzyme in PPP and platelets, wherein the isoform XO showed the highest activity and XD the lowest one (Figure 3). However, in the case of the intermediate isoform higher activity is found in platelets more than in plasma, which indicates a more rapid conversion of the intermediate isoform XD. According to Dolegowska et al., XOR activity and its isoforms were tested in plasma in patients after renal transplantation divided into three groups: EGF: early, SGF: slow, DGF: delayed graft function. XO showed increased activity and XOR in all groups at 1 and 5 minutes after transplantation. XD activity increased in the groups of SGF and DGF also at 1 and 5 minutes. The highest activity was found in the isoform XD (having an antioxidant effect) and the lowest activity was found in isoform XO (having oxidative effects). This may mean that, in severe conditions of oxidative stress, such as organ transplant, comes to increased activity of the enzyme, leveling oxidative stress. This explains the highest activity XO isoforms in normal individuals not exposed to oxidative stress [3]. Kim et al., in turn, demonstrated that patients suffering from lung cancer survival are associated with the activity of XOR. Survival in patients with a higher activity of oxidoreductase is longer than that in patients with a lower activity of the enzyme [16]. Linder et al. achieved similar results in the case of serous ovarian cancer. They showed that a decreased XOR activity is associated with a worse prognosis of patients suffering from this disease, especially those with unfavorable prognostic profile [17].

Boban et al. studied the activity of the XOR and XO and XDH in patients with spontaneous hypertension and patients on dialysis. This study has shown that the total XOR activity was higher in the patients suffering from spontaneous hypertension, as compared to patients on dialysis. Also in this group the highest activity of XDH is showed, in relation to the control or dialysis.

On the other hand, the activity of XO, which mainly contributes to the production of ROS, was the highest in the dialysis patients [18]. However, in another study, Linder et al. showed a lower XOR activity in the 62% of colon cancer tissues and the enzyme activity detectable in 22% as compared to healthy tissue. Also the degree of differentiation of tumor cells has an effect on the activity of XOR, which is significantly higher in cells having a large degree of variation. This means that the activity of the XOR is associated with the degree of differentiation of tumor and its advancement in the case of colon cancer and can be a prognostic factor [19].

In our study, there was no correlation between the activity of XOR and its isoforms and the age of subjects. However, XOR activity in the case of platelet-poor plasma result was close to statistical significance. This means that in order to be able to clearly put such a request it is necessary to increase the test group. No association was found between the activity of XOR and gender of healthy subjects. Decker and Levinson XOR activity assays by RIA showed a higher activity of the enzyme in male rats [20]. Results achieved by us in relation to the analysis of other authors confirm the physiological significance of xanthine oxidoreductase and demonstrate the utmost importance and the need for this type of research in the context of the prognostic significance of XOR in the case of many types of chronic diseases such as kidney disease or tumors of various types.

## 5. Conclusions

The healthy volunteers showed the highest activity isoform XO (prooxidant) and the lowest isoforms XD (antioxidant), which indicates a slight oxidative stress in people tested and confirmed physiological effects of XOR. There were significant differences in the activity of XOR and its isoforms in PPP and platelets. There was no correlation between the activity of XOR and the age and gender of healthy volunteers.

## Figures and Tables

**(a):** 

Parameters characterizing healthy volunteers		Female	Male	*P* value
Number of healthy volunteers	70	48	29	NS
Age > 30 years	20	14	6	<0.001
Age < 30 years	49	34	15	<0.001
Smoking	10	8	2	NS

NS: no statistical significance.

**(b):** 

Parameters characterizing healthy volunteers	
Heart diseases	2
Hypertension	6
Diabetes	1
Kidney disease	6
Vascular disease	6
Related allergological diseases	19

NS: no statistical significance.

**Table 2:** Xanthine oxidoreductase activity in PPP and platelets, in the individual isoforms (mean ± SD).

Variable activity [mU/mL]	Mean ± SD	Minimum; maximum
XDH PPP	38.7 ± 16.2	11.8; 99.1
XDH platelets	6.9 ± 3.6	1.6; 18.03
XDO PPP	39.1 ± 19.5	1.6; 116.2
XDO platelets	11.6 ± 5.1	1.5; 27.3
XO PPP	49.6 ± 18.9	10.7; 96.6
XO platelets	12.2 ± 5.37	0.65; 25.2
Xanthine oxidoreductase activity in PPP	42.5 ± 18.8	1.57; 116.2
Xanthine oxidoreductase activity in platelets	10.2 ± 5.3	0.65; 27.3

SD: standard deviation.

**Table 3:** XOR reference values and its isoforms in PPP and platelets, in healthy volunteers.

Reference values and its isoforms XOR PPP and platelets in healthy control	
XOR PPP	10.93–23.58
XOR platelets	3.28–23.58
XD PPP	14.05–89.86
XD platelets	2.46–17.22
XDO PPP	5.89–88.28
XDO platelets	2.75–24.98
XO PPP	19.06–86.90
XO platelets	3.55–24.87
